# Effects of a Novel Bovine Lactoferrin-Derived Peptide on the Intestinal Morphology and Intestinal Flora in Rats

**DOI:** 10.3390/microorganisms13050975

**Published:** 2025-04-24

**Authors:** Tianle Huang, Huan Yang, Yang Zhao, Haiyue Cui, Xiaoxi Qi, Liguang Miao

**Affiliations:** Institute of Special Animal and Plant Sciences, Chinese Academy of Agricultural Sciences, Changchun 130112, China; 82101225660@caas.cn (T.H.);

**Keywords:** bovine lactoferrin, intestinal morphology, intestinal flora

## Abstract

Bovine lactoferrin-derived peptide LF-MQL was administered to healthy rats to assess its effects on growth parameters and gut morphology. Faecal samples were analysed by 16S rRNA high-throughput sequencing to investigate the modulatory effects of LF-MQL on the composition and diversity of the gut microbiota. The results showed that both experimental groups maintained intact intestinal organization. Notably, supplementation with LF-MQL significantly increased the length of small intestinal villi compared to the control group (*p* < 0.05), and an improvement in the structural organization of the villi, including a more ordered and compact arrangement, was observed. These morphological findings suggest that there are no adverse effects associated with LF-MQL administration. In addition, administration of LF-MQL modulates the functional activity of the gut microbiota and regulates their involvement in host-related metabolic pathways, thereby improving gut homeostasis. These findings provide a theoretical basis for evaluating the safety of bovine lactoferrin peptides in food and pharmaceutical applications.

## 1. Introduction

Gut flora plays an important role in host health and disease [1], and the composition and products of intestinal microorganisms have a strong influence on the immune response [2]. Under normal conditions, the intestinal flora maintains a dynamic equilibrium and interacts with the host and can be viewed as a bacterial organism in the body with a variety of functions [3]. Changes in the intestinal flora occur in the course of a wide range of diseases that disrupt the fine balance of the intestinal flora, leading to changes in its composition and make-up [4].

Gut microorganisms can degrade food that the host cannot digest; *Trichoderma* spp. and *A. tumefaciens* spp. can ferment carbohydrates to produce volatile fatty acids, while degradation of plant aromatic compounds is another source of volatile fatty acids in the large intestine, and flavonoids and lignin complexes can be converted to acetate and butyrate by the interactions of several species of bacteria [5].

The enormous genetic diversity of gut microbes can provide many biological activities that are lacking in the host. Microbes colonize the mammalian host immediately after birth. Many resident bacteria are adapted to the intestinal environment and interact in complex ways with other bacteria and host ecological niches to obtain nutrients. The composition of the microbiota is highly dependent on the nutritional requirements of individual bacteria and is highly variable at different locations in the gut [6]. The small intestine is rich in mono- and disaccharides as well as amino acids that support the growth of *Aspergillus* and *Lactobacillus* bacteria [7]. In contrast, the vast majority of available sugars in the colon are dietary and host-derived complex carbohydrates that are indigestible by the host. Bacteriophages and Clostridia contain enzymes that can break down complex polysaccharides, including fibres and mucins, and use them as a source of energy. Therefore, bacteria belonging to the orders Bacteroidetes and Clostridia are the major populations in the large intestine. Neonatal mammalian intestinal microorganisms are mainly derived from the mother and the external environment. After birth, aerobic and anaerobic bacteria first colonize the gut in neonatal rats, which changes to an anaerobic environment as oxygen is consumed.

Taxa including *Anabaena*, *Lactobacillus*, *Eubacterium* spp. via mucus, *Clostridium* spp., and *Klebsiella* spp. were more abundant in the intestinal flora of pre-weaned rats compared to post-weaned rats. The predominant gut microorganisms in rats in the first year after weaning were the phylum Thick-walled Bacteria and the phylum Anaplasma, with mucinophilic Acromycetes accounting for a large portion of the microbial composition [8].

Modulation of gut microbes has emerged as a new approach to maintaining health and boosting immunity, and a series of studies have demonstrated that modulation of the gut flora using certain agents, such as Chinese herbs, probiotics, and certain protein peptides, can result in varying degrees of improvement in the gut health of test animals [9,10,11].

Lactoferrin, a glycoprotein found in whey, is a multifunctional transferrin glycoprotein that is widely distributed in the exocrine fluids of the human body. Due to its strong iron-binding ability, LF can compete with harmful intestinal bacteria for iron ions, resulting in an antibacterial effect and regulation of intestinal flora [12]. The main lactoferrin commonly used in research is human and bovine lactoferrin, with the highest concentration of lactoferrin found in the human colostrum [13].

Lactoferrin is one of the components of the human immune system. The structure of lactoferrin contains a polypeptide chain that contains about 700 amino acids and two homologous globular structural domains, the N and C loops, where the N loop corresponds to amino acid residues 1 through 333 and the C loop corresponds to amino acid residues 345 through 692, and the two domains are connected at the ends by a short piece of alpha-helical chain, with two subdomains in each loop, and contains an iron binding site and a glycosylation site [14].

Several studies have shown that lactoferrin has a modulating effect on intestinal flora and can reduce tissue weight, visceral obesity, and hepatic lipid accumulation in metabolically disordered mice through this effect and adjust levels of glucose and pyruvate metabolism in the intestinal flora of mice [1].

Based on extensive research on BLFcin, our team developed a novel peptide with effective immunomodulatory and antimicrobial properties, named LF-MQL, with a molecular weight of 10KD [15]. The antimicrobial and immunomodulatory properties of LF-MQL have been demonstrated in previous studies, where it was found that LF-MQL reduced the mortality rate of chicks infected with Salmonella dysentery and mice infected with Salmonella typhimurium without causing toxicity to the mice and enhanced the in vivo and in vitro immune responsiveness of the mice [16,17]. The aim of this study was to detect changes in intestinal morphology and intestinal flora in rats under the influence of LF-MQL.

## 2. Materials and Methods

### 2.1. Materials

Twenty specific pathogen-free male SD rats (3 weeks old) were randomly divided into control group and LF-MQL test group, 10 rats in each group, each weighing about (75 ± 3) g. The rats were purchased from Liaoning Changsheng Biological Co. (Benxi, China). The room temperature was (25 ± 2) °C, humidity was 50%, and the light–dark cycle was 12:12. The experimental animal feed was the rat feed provided by the above company. Oral tablets (1 mg of LF-MQL per 1 g of oral tablet) were prepared with LF-MQL samples and dextrin.

### 2.2. Animal Grouping

The experiment was divided into 3 phases, i.e., the adaptive feeding phase, the intervention phase, and the sampling phase.

Adaptive feeding phase: all rats were fed with the standard training diet for a 1-week acclimatisation period.

Intervention phase (2 weeks): at the end of the acclimatisation feeding, the rats were randomly assigned into a control group and an MQL test group, with 10 rats in each group. The MQL test group was fed 1 g of LF-MQL oral tablets per day, and the control group was fed 1 g of dextrin oral tablets per day. The rats had free access to feed and water during the test period. The body weight, feed intake, and water intake of the rats were recorded daily and the faeces of the rats were collected at the end of the test.

Sampling phase: 6 rats were selected from each group for sampling. Water was allowed to be added before the start to empty the gut of residual food. Subsequently, the rats were anaesthetised using chloroform, a portion of small intestinal tissue was removed and fixed in a centrifuge tube containing 4% paraformaldehyde solution, and faeces were collected from the rectum of the rats.

### 2.3. Small Intestine Sections Observation (Using the Eclipse Ci-L Microscope)

After the rat small intestine segments were fixed with 4% paraformaldehyde for 48 h, small intestine tissue blocks (15 mm × 15 mm × 15 mm) were taken for tissue sectioning and staining tests as follows.

### 2.4. Faecal Sample Collection

After the rats were anaesthetised and the abdominal cavity was opened to remove the small intestinal samples, the faeces were discharged by squeezing the rectum and immediately collected into sterile freezer tubes and quickly frozen on dry ice. The faeces were stored in a refrigerator at −80 °C before the test.

### 2.5. Intestinal Flora Testing

Faecal samples were kept on dry ice and subjected to intestinal flora DNA extraction, and genomic DNA from the samples was extracted using the Magpure Soil DNA LQ Kit (Magan, Aurora, ON, Canada) according to the instructions. The concentration and purity of DNA was checked by NanoDrop2000 (Thermo Fisher Scientific, Waltham, MA, USA) and agarose gel electrophoresis, and the extracted DNA was stored at −20 °C. The extracted DNA was stored at −20 °C. The extracted genomic DNA was used as a template for PCR amplification of the bacterial 16s rRNA gene using specific primers with Barcode and the Takara Ex Taq high-fidelity enzyme, and the 16s rRNA gene was amplified using the universal primers 343F (5′-TACGGRAGGCAGCAG-3′) and 798R (5′-CCGTCAATTCMTTTRAGTTT-3′). Amplification of the V3–V4 or (V4–V5) variable region of the 16s rRNA gene for bacterial diversity analysis was conducted.

### 2.6. 16s Diversity Sequencing Analysis Method

The raw data were in FASTQ format. After the data were downloaded from the machine, the raw data sequences were firstly cut off the primer sequences using the Cutadapt (1.9.1) software. Then, using DADA2, the qualified double-ended data from the previous step were selected as representative sequences of each ASV according to the QIIME 2 (2020.11) software package, and all the representative sequences were annotated against the Silva (version 138) database. Species comparison annotations were analysed using the default parameters of the q2-featue-classifier software.

Alpha and beta diversity analyses were performed using the QIIME 2 software. The alpha diversity of the samples was assessed using alpha diversity including the Chao1 index and the Shannon index. Unweighted Unifrac principal coordinate analysis (PCoA) was performed to assess the beta diversity of the samples using the unweighted Unifrac distance matrix computed in R (4.3.2). The unweighted Unifrac distance matrix was used to assess the beta diversity of the samples. The ANOVA/Kruskal–Wallis/T-test/Wilcoxon statistical algorithms based on the R package were used for analysis of variance. Species abundance spectra were analysed for differences using LEfSe.

## 3. Results

### 3.1. Effects of Bovine Lactoferrin Peptide LF-MQL on Body Weight, Feed Intake, and Water Intake in Rats

The effects of LF-MQL intake on the health of the rats as well as on the diet digestion and absorption of the rats were reflected by recording the differences in body weight gain, feed intake, and water intake. The index changes of the rats throughout the test period are shown in Table 1. The increase in body weight of the rats in all groups indicates the growth of the rats after the intake of LF-MQL and dextrin. Increased body weight gain was observed in the MQL group as compared to the control group. Surface LF-MQL significantly increased the growth level of rats. Water intake of the two groups of rats were not significantly different from each other (*p* > 0.05). However, the average daily weight gain and average daily feed intake were significantly higher in the experimental group compared to the control group (*p* ≤ 0.05).

### 3.2. Effects of LF-MQL on Intestinal Morphology

**Figure 1:**
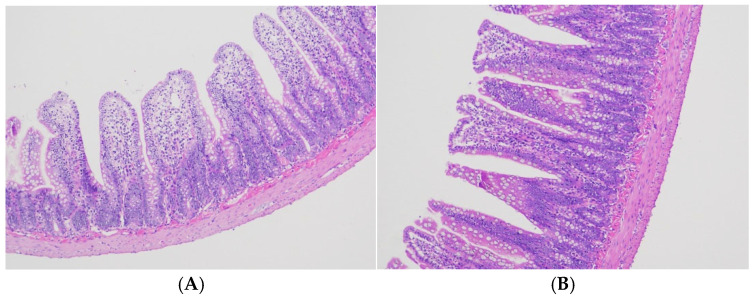
Analysis of morphological changes in the small intestine of a rat. (**A**) Control group. (**B**) MQL group.

Histological pictures (Figure 1) showed that the MQL group had better intestinal tissue integrity and longer, tighter, and more aligned intestinal villi compared with the control group. The above results indicated that LF-MQL significantly affected the length of small intestinal villi and changes in intestinal morphology. LF-MQL helped to improve the intestinal morphology and increase the contact area of the small intestine with nutrients, and the length of intestinal villi was increased and the depth of crypts was decreased in the MQL group compared with the control group (Table 1) [18].

### 3.3. Quality Assurance of Intestinal Flora Samples

**Figure 2:**
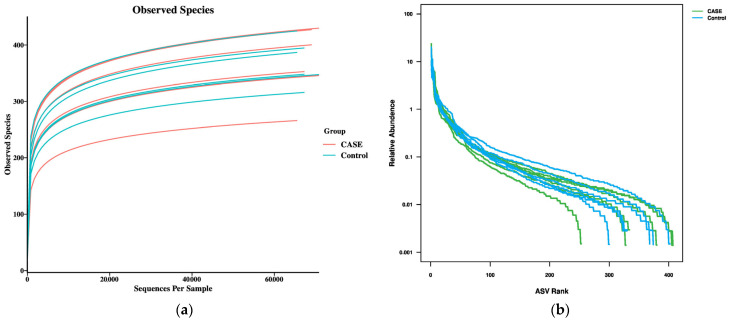
Quality identification of rat intestinal microbiota samples. (**a**) Sob dilution curves; (**b**) rank–abundance curves.

In order to evaluate whether the amount of sequencing data in this experiment is reasonable or not, the Sobs dilution curve and rank–abundance curve were constructed by dividing the non-repetitive sequences by ASV using 97% similarity as the threshold. The Sobs dilution curve showed (Figure 2a) that when the amount of sequencing data was small, the increase in the number of species detected in the faecal samples increased dramatically with the gradual deepening of the sequencing depth. At a cumulative sequencing data volume of 15,000, the Sobs curves of the two groups of experimental samples gradually flattened out. The above results indicate that the amount of sequencing data in this experiment is reasonable and the sequencing depth is sufficient. The rank–abundance ranking curve (Figure 2b) reflects the levels of species abundance and species evenness, in which species abundance is proportional to the range of the horizontal curve and species evenness is proportional to the smoothness of the curve. As the width in the horizontal direction deepens, the range of the curve on the horizontal axis gradually becomes larger, and the curve is flattened (indicating smoothness), indicating relatively high species abundance and evenness. In summary, the sequencing depth of the two groups of samples in this experiment is sufficiently reasonable to reflect the information of most species in each group. In addition, the abundance and homogeneity of microorganisms in the rat gut flora were good.

### 3.4. Changes in the Diversity of Gut Flora

#### 3.4.1. Alpha-Diversity Analysis

Alpha-diversity analyses reflect the diversity of species in a single sample, and the main metrics analysed include the Chao1 index, the Ace (abundance-based coverage estimation) index, the Shannon index, and the Simpson diversity index. The Ace index (which evaluates the number of ASVs of a community) and the Chao1 (which estimates the total number of species) reflect the abundance of the colony, and the magnitude of each is positively and inversely proportional to the colony abundance and proportional to community diversity. The Shannon index (an estimate of the diversity of the sample community) and the Simpson index (which quantifies the diversity of the community in a given area) reflect community diversity and are proportional and inversely proportional to community diversity, respectively. LF-MQL intake improved the abundance and diversity of rat gut flora compared to the control group (Table 2). There was no significant difference in the Shannon and Simpson indices between the experimental and control groups, indicating that to some extent, the diversity of microbial communities was similar in both groups (*p >* 0.05). There were also no significant changes in the Ace index and Chao1 index in both groups, reflecting that feeding LF-MQL did not increase the number of ASVs and the total number of species in the community (*p* > 0.05).

#### 3.4.2. Beta-Diversity Analysis

**Figure 3:**
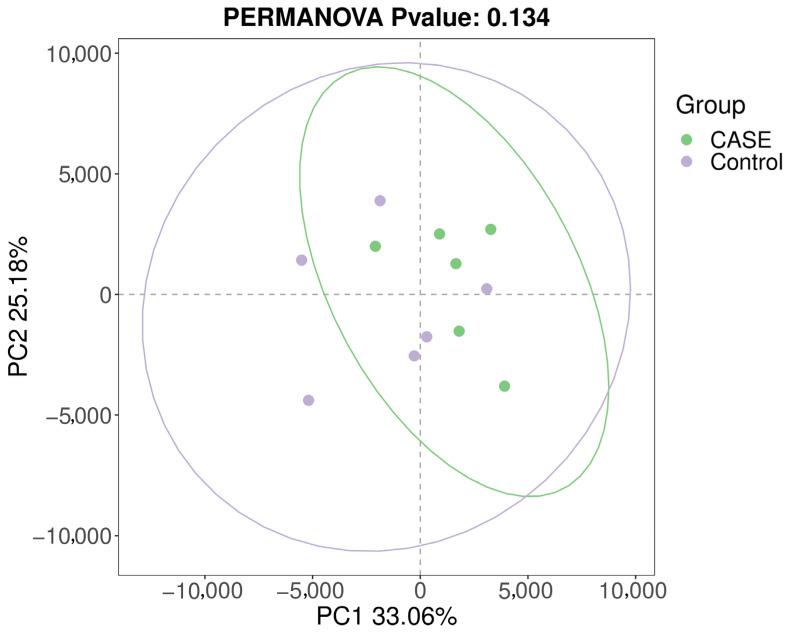
Intergroup analysis of PCoA based on weighted Unifrac distance.

**Figure 4:**
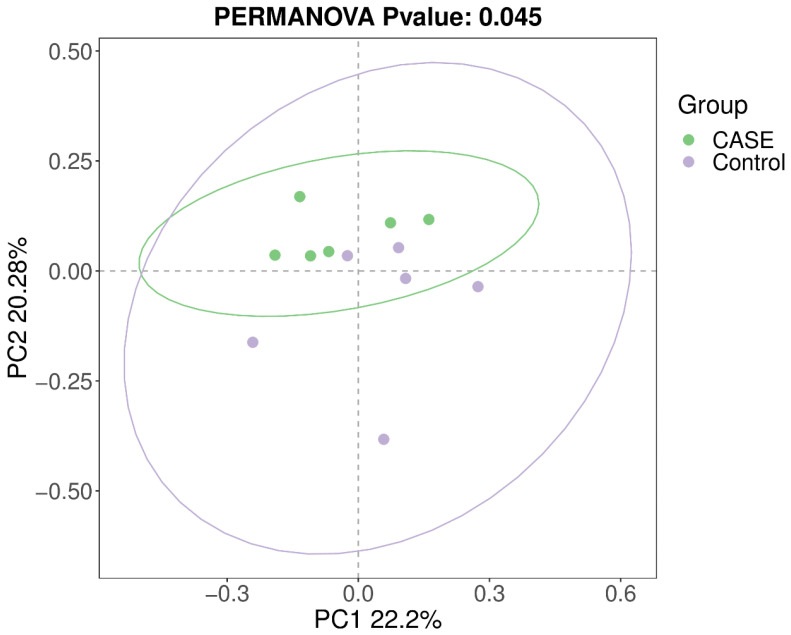
Intergroup analysis of PCoA based on unweighted Unifrac distance.

Between-group differences in gut microbial community composition across samples were explored using β-diversity analysis. In this study, intergroup differences in gut microbial community composition between the two groups of samples were analysed at the ASV level based on weighted single cleavage distances and unweighted single cleavage distances from principal coordinate analysis (PCoA) and the results are shown in Figure 3 and Figure 4.

### 3.5. Analysis of the Composition of Gut Flora

**Figure 5:**
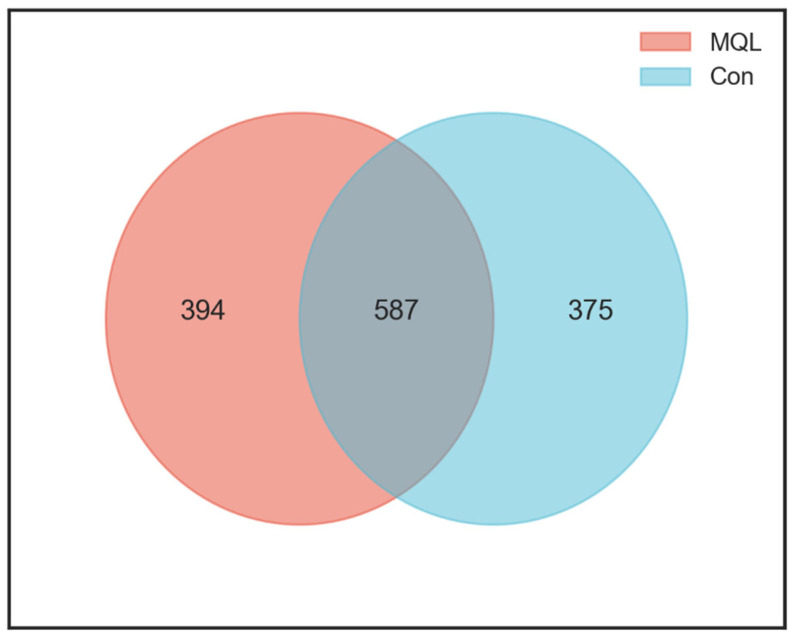
Venn diagram of ASVs.

The Wayne plots obtained from the clustering analysis of ASVs can be used to count the number of species shared by rats from different experimental groups and the uniqueness of individual samples, which helps to show the overlap and similarity of species composition in the intestinal flora samples of the two groups, and the results of the analyses are shown in Figure 5. There was a total of 587 identical ASVs in the two groups of rats, of which 394 were unique to the MQL group, and 375 were unique to the Con group. The number of species shared by the two groups of rats is shown in Figure 5. Thus, LF-MQL intake increased the abundance of rat gut flora.

**Figure 6:**
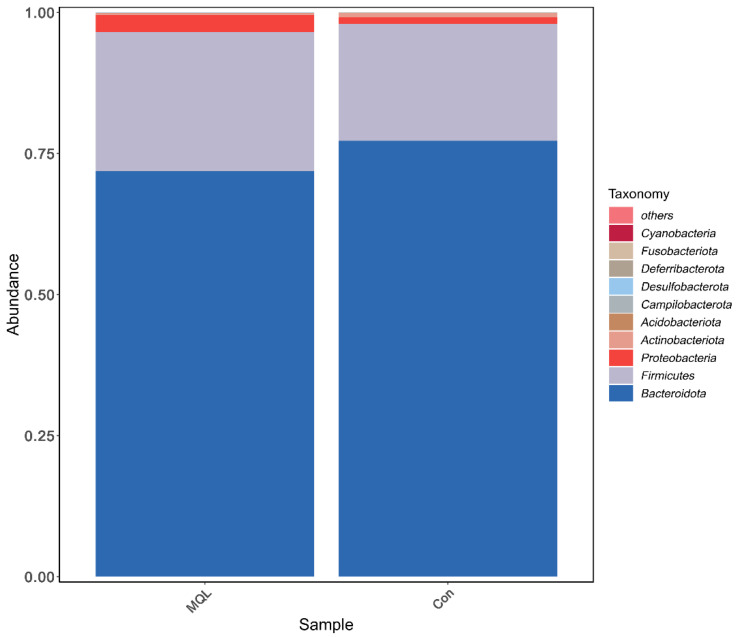
Histogram of community structure at the phylum level.

At the phylum level, the dominant phyla in the group samples were Bacteroidota, Firmicutes, and Proteobacteria. The six phyla with the highest relative abundance contained more than 95% of the samples in each group; in addition, the relative abundance of the dominant phyla was altered by LF-MQL intake, and the relative abundance of the major phyla was altered by LF-MQL intake. See Figure 6 and Table 3 for details.

**Figure 7:**
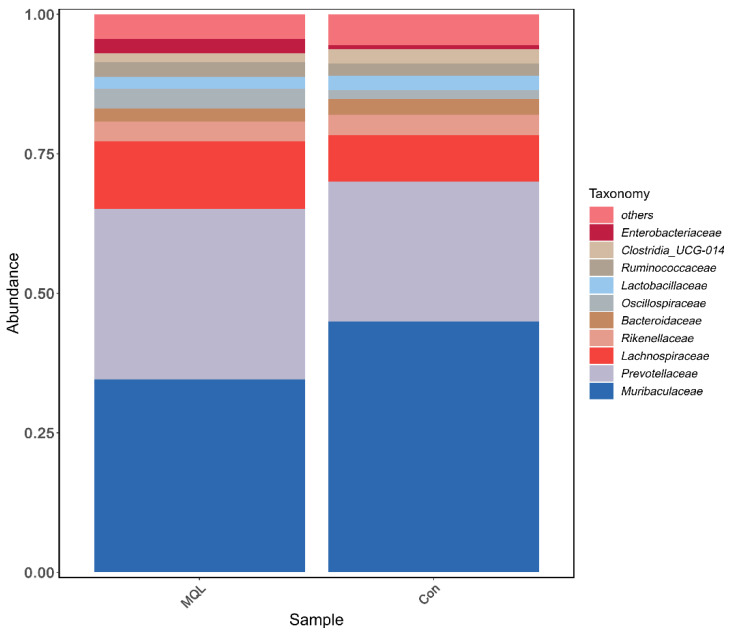
Histogram of community structure at the family level.

Relative abundance at the family level: The top 15 rat gut microorganisms in terms of relative abundance at the family level were Muribaculaceae, Prevotellaceae, Lachnospiraceae, Oscillospiraceae, Rikenellaceae, Ruminococcaceae, Lactobacillaceae, Bacteroidaceae, Clostridia_UGG-014, Enterobacteriaceae, [Eubacterium]_coprostanoligenes_group, Tannerellaceae, Corynebacteriaceae, Peptostreptococcaceae, and Marinifilaceae. The highest abundance in each group was Muribaculaceae, with an abundance of 37.14% in the LF-MQL test group and 39.74% in the control group. See Figure 7 and Table 4.

**Figure 8:**
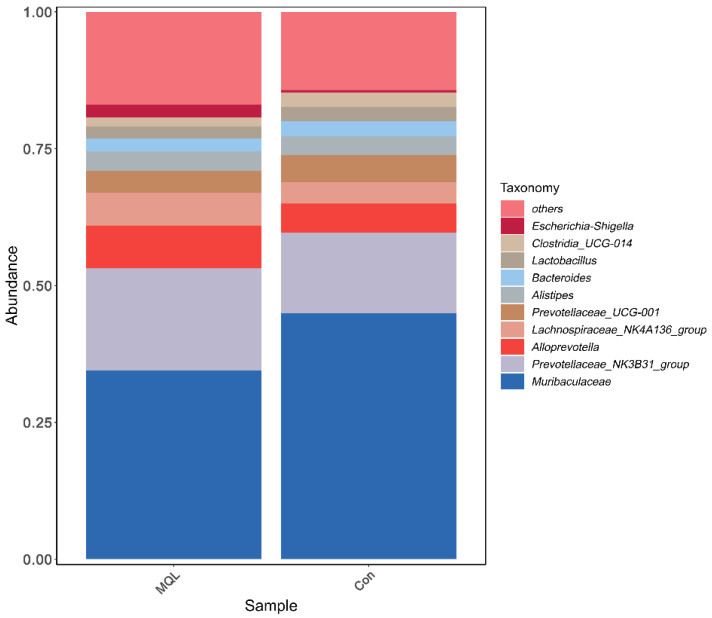
Histogram of community structure at the genus level.

Relative abundance at the genus level (genus): The dominant groups of rat gut microorganisms at the genus level were mainly as follows: Muribaculaceae, Prevotellaceae_NK3B31_group, Lachnospiraceae_NK4A136_group, Alloprevotella, Alloprevotella Prevotellaceae_UCG-001, Alistipes, Lactobacillus, Bacteroides, Clostridia_UCG-014, Escherichia, Shigella, Ruminococcus, Roseburia, [Eubacterium]_coprostanoligenes_group, [Eubacterium]_siraeum_group, and Lachnospiraceae_UCG-001. See Figure 8 and Table 5.

### 3.6. LEfSe Analysis of Differential Intestinal Flora

**Figure 9:**
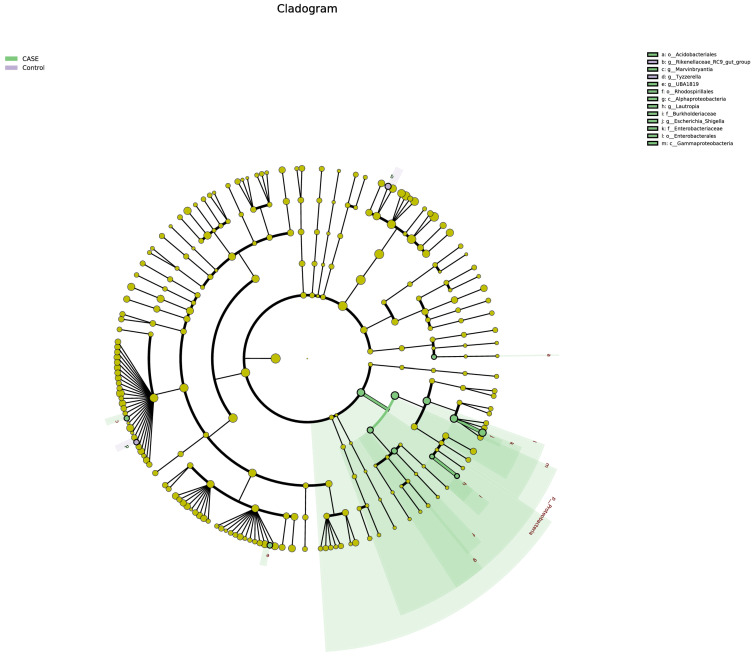
Evolutionary classification tree diagram of gut microorganisms.

The differential flora between the two groups was analysed by LEfSe (Linear discriminant analysis Effect Size). Setting the LDA greater than the base set value of 2, it was determined that the species difference had a significant effect. Figure 9 demonstrates the evolutionary branching diagram of rat intestinal microorganisms from the phylum level to the species level, and the analysis shows that at the phylum level, the Proteobacteria abundance in the test group was significantly higher than that of the control group (*p* < 0.05); at the phylum level, Enterobacterales abundance in the test group was significantly higher than that of the control group (*p* < 0.05); and at the genus level, Escherichia spp., Muribaculum, UBA1819, Marvinbryantia, and Lauteropia spp. had significantly (*p* < 0.05) higher relative abundance in the test group compared to the control group.

### 3.7. Prediction of Biological Functions of Intestinal Flora

The 16sr sequencing data (GOG analysis and KEGG analysis) were analysed using the PICRUSt2 (2.5.0) functional software to predict the functional gene composition of each group of samples, thus analysing the main biological functions designed for the different subgroups as well as the functional differences between them [19].

#### 3.7.1. COG Functional Analysis

**Figure 10:**
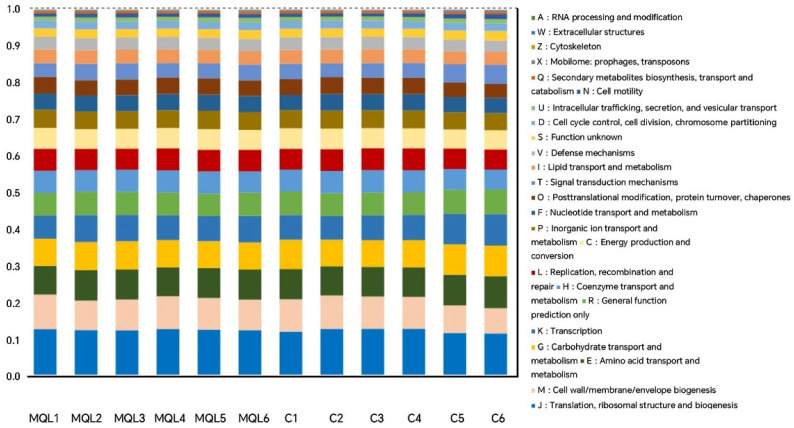
Functional relative abundance bar chart based on COG analysis. MQL1~MQL6, MQL group; C1~C6, control group.

The results of the functional classification and relative abundance analysis of COG are shown in Figure 10. A total of 3951 COG functional units were detected in this assay, which belonged to 21 functional categories. Compared with the control group, the MQL test group up-regulated the rat gut microbial communities in J: translation, ribosomal structure, and biogenesis, M: cell wall/membrane/envelope biogenesis, H: coenzyme transport and metabolism, L: replication, recombination and repair, C: energy production and conversion, P: inorganic ion transport and metabolism, F: nucleotide transport and metabolism, O: post-translational modification, protein turnover, and chaperones, I: lipid transport and metabolism, V: defence mechanisms, Q: secondary metabolite biosynthesis, transport, and catabolism, A: RNA, E: amino acid transport and metabolism, G: carbohydrate transport and metabolism, K: transcription, R: general function prediction only, T: signal transduction mechanisms, S: function unknown, N: cell motility, X: mobilome, prophages, and transposons, and W: down-regulation of extracellular structures.

The above results suggest that LF-MQL intake can improve the intestinal health of rats by altering the physiological functions of intestinal flora.

#### 3.7.2. Prediction of KEGG Biological Function

To further explore the functional changes of rat gut microbial communities after LF-MQL intervention, all sequencing data were analysed using KEGG analysis to predict primary functional pathways, secondary subfunctional pathways, and tertiary metabolic pathways.

#### 3.7.3. KEGG Tier Functional Prediction

The results of the comparative analysis of the relative abundance of biological functions predicted by the first tier of function are shown in Table 6. The database contains six major biometabolic pathways: metabolism, genetic information processing, environmental information processing, human disease, cellular processes, and organismal systems. The relative abundance of metabolic functions was the highest in both groups, and LF-MQL intake reduced the relative abundance of gut flora in the cellular processes and environmental information processing pathways compared to the control group.

#### 3.7.4. Analysis of KEGG Second-Level Subfunctional Pathways

**Figure 11:**
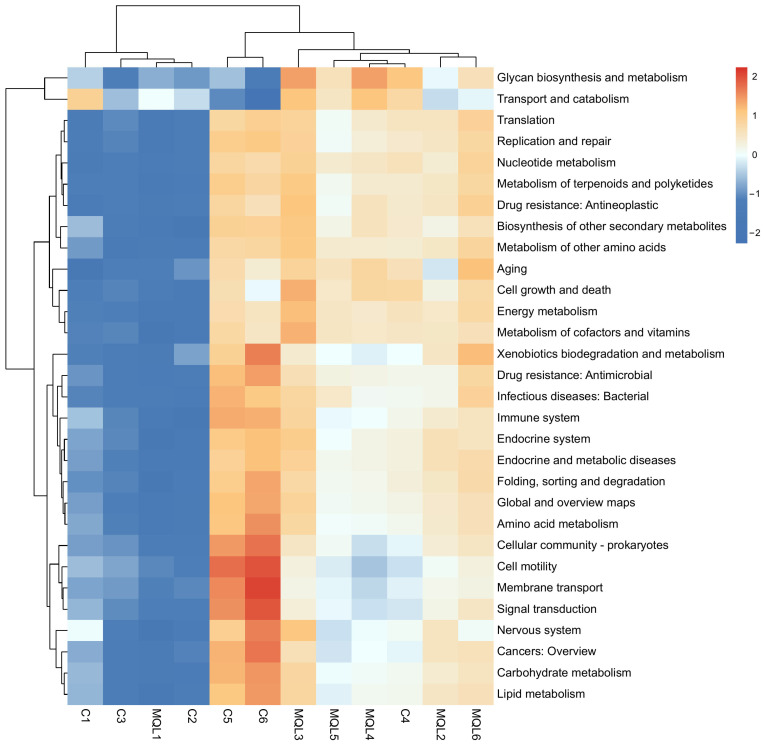
KEGG heat map. C1~C6, control group; MQL1~MQL6, MQL group.

Further correlation heatmap analysis was performed on the predicted second-tier subfunctional pathways and the results are shown in Figure 11. The top 10 pathways predicted in terms of abundance are global and overview maps, carbohydrate metabolism, amino acid metabolism, energy metabolism, cofactor and vitamin metabolism, translation, replication and repair, nucleotide metabolism, and membrane transport, which are mainly involved in a range of functional pathways such as energy metabolism, amino acid metabolism, and transmembrane transport and a series of other functional pathways. In order to better reflect the differences and similarities between the predicted results of KEGG secondary functions in the two groups, correlation heatmaps of the top 30 functional pathways in relative abundance were drawn. It can be clearly observed that the variability of KEGG function prediction results between the groups is small.

#### 3.7.5. Analysis of KEGG Tertiary Metabolic Pathways

In the final search of KEGG tertiary metabolic pathways, a total of 300 pathways were labelled in this experiment and the top 30 pathways in the clustering number in the clustering difference were compared, and the results are shown in Table 7. Compared to the control group, intake in the MQL group down-regulated the pathways of biosynthesis of valine, leucine, and isoleucine, the two-component system, tryptophan metabolism, toxoplasmosis, the thyroid hormone signalling pathway, and the biosynthesis of tetracycline. In addition to this, the number and proportion of genes clustered in the top 30 pathways were significantly up-regulated in the MQL group compared to the control group.

## 4. Discussion

Current research suggests that the number of microorganisms living in the intestines of monogastric animals is about 1014. There are at least 10 times the number of microorganism cells compared to the number of host cells, and there are about 500–1000 populations of these microorganisms whose species include bacteria, fungi, archaea, and protozoa. These microorganisms can reach 100–200 times the host genome in terms of the number of genes encoded. Thus, gut microbes can participate in host physiological processes by providing biological activities that the host lacks. Microorganisms colonize the mammalian host immediately after reproduction and acquire nutrients within the host through a number of complex interactions with other microbial ecological niches. To some extent, the composition of the microbiota is largely determined by the nutritional requirements of a particular individual bacterium, and variations are highly correlated with differences in gut location [20].

The small intestine is rich in mono- and disaccharides as well as amino acids, making it suitable for the growth of Proteus and *Lactobacillus* [5]. The gut microorganisms of a mammal at birth are mainly derived from its mother and the external environment. After birth in neonatal rats, aerobic and anaerobic bacteria gradually colonize the gut, which changes to an anaerobic environment as oxygen is consumed. Compared with the post-weaning period, the gut flora of pre-weaned rats had a higher abundance of Bacteroides immitis, *Lactobacillus*, *Eubacterium transmucilaginosum* spp., *Clostridium* spp., and *Klebsiella* spp. The predominant gut microorganisms in rats in the first year after weaning were Firmicutes and Bacteroides, with Akkermansia muciniphila accounting for a large portion of the microbial composition. Microbial community diversity increased along the longitudinal axis of the rat GI tract, with the stomach and duodenum having similar levels of diversity as the large intestine. In addition to *Lactobacillus*, a lactic acid-producing Turicibacter dominated the rat GI tract. The abundance of lactic acid-producing bacteria was relatively high in the upper GI tract, resulting in high levels of lactic acid in the stomach and small intestine. The pattern of fermentation in the lower GI tract is quite different from that in the upper GI tract, where an increased production of volatile fatty acids, especially butyrate, can be observed, and Lachnospira and Ruminococcus, which produce volatile fatty acids, constitute the bulk of the microbiota of the lower GI tract [21].

Gut microbes play an important role in participating in nutrient metabolism, inhibiting pathogenic microbes, and promoting intestinal mucosal immunity. Gut microorganisms can degrade food that cannot be digested by the host; *Trichoderma* spp. and *Ruminalia* spp. can ferment carbohydrates to produce volatile fatty acids and can convert flavonoids and lignin complexes to acetate and butyrate in the interactions of a variety of bacteria. Proteins, amino acids, peptides, and endogenous secreted proteins in food are sources of nitrogen essential for the gastrointestinal tract and commensal bacteria during growth, development, and metabolism. Microbial diversity has a positive correlation with proteins in food, and the quantity and quality of proteins can positively influence microbial diversity. Proteases and peptidases produced by gut microflora are able to degrade proteins into substances such as peptides and amino acids that can be utilized by the body [22]. Gut microorganisms inhibit the colonization of pathogenic microorganisms by competing with them for nutrients and adhesion sites. Lactic acid-producing and fatty acid-producing bacteria in the intestinal flora produce lactic acid and short-chain fatty acids as a means of lowering the pH in the intestinal tract and inhibiting the growth of pathogenic bacteria. Gut microbes help maintain the integrity of the intestinal epithelium and promote the formation of the intestinal mucosal immune system. Gut microbes promote the development and maturation of immune organs [23].

The results of the 14-day feeding test on rats showed that the addition of LF-MQL to the diet significantly increased the average daily weight gain and average daily feed intake of rats. The results showed that the LF-MQL samples could promote the growth and development of rats to a certain extent, and the increase in the daily feed intake of rats indicated that the LF-MQL samples had good palatability, and at the same time, it could also increase the utilization rate of the feeds, promote feed intake, and facilitate the nutrients to enter into the organism so as to better digest and metabolize them, thus promoting the growth of mammals. This suggests that LF-MQL can affect the growth performance of rats by improving the digestion and absorption of nutrients.

The growth and development of the animal organism is also directly affected by the efficiency of digestion and absorption of nutrients. The morphology and structure of intestinal tissues, especially the height of the intestinal villi and the depth of the crypts, have a direct influence on the digestion and absorption of nutrients in the animal organism. Because of this, the ratio of the height of the small intestinal villi to the depth of the crypts, i.e., the villus-to-crypt ratio, not only serves as an important indicator reflecting the morphology of intestinal development but can also indicate that the digestive capacity of the animal improves when this ratio is elevated [24].

Lactoferrin and lactoferrin-derived peptides exert a positive effect on intestinal health, which has been confirmed in many studies. In this study, the height of small intestinal villi in the test group was significantly higher, the depth of crypts was significantly lower, the villus-to-crypt ratio was significantly higher, and the small intestinal villi were more abundant, indicating that LF-MQL can promote intestinal development and improve the digestion and absorption of nutrients in rats.

Differences in species richness and diversity between the experimental and control groups were assessed by α-diversity analysis, in which the ACE index and the Chao1 index reflected species richness, and the Shannon index and Simpson’s index were used to assess species diversity. The results of this study showed that LF-MQL intake improved the abundance and diversity of rat intestinal flora compared with the control group; there was no significant difference in the ACE index, the Chao1 index, the Shannon index, and Simpson’s index between the experimental and control groups, which indicated that to a certain extent, the diversity of the microbial communities was similar in the two groups.

Species annotation of representative ASV sequences was performed by the 97% similarity threshold, and the differences in community composition between the experimental and control groups were counted at six taxonomic levels, from phylum to species. The results showed that the phyla Bacteroidota, Firmicutes, and Proteobacteria dominated, and the three together exceeded 95% of the total sequences of the flora. This is consistent with Tang et al.’s study on the study of the intestinal flora of rats within one year of weaning [22], and from the point of view of the hierarchical specificity characteristics, at the level of the phylum, in the experimental group, Firmicutes and Bacteroidota had no significant change in abundance; at the family level, the relative abundance of Prevotellaceae, the most abundant microorganism among the Bacteroidetes, was significantly higher in the LF-MQL test group compared to the control group (*p* = 0.422), and it is important for the degradation of plant nonfibrous polysaccharides and proteins [25]; and at the genus level, the relative abundance of Esherichia-Shigella, Muribaculum, UBA1819, Marvinbryantia, and Lautropia in the LF-MQL test group was significantly higher than that of the control group, in which Muribaculum can increase insulin sensitivity, reduce host obesity by producing SCFA, promote lipolysis and fatty acid oxidation, and inhibit cholesterol synthesis in the liver.

LF-MQL intervention significantly remodelled the intestinal flora structure, as evidenced by the enrichment of metabolically beneficial bacteria and the inhibition of pro-inflammatory genera. This flora remodelling may reduce the risk of associated diseases by enhancing short-chain fatty acid synthesis and inhibiting inflammatory pathways. However, the specific mechanism of interactions between flora function and host metabolism still needs to be verified by joint multi-omics analysis.

## 5. Conclusions

In this study, LF-MQL was used to intervene on healthy rats. The results showed that both groups of rats gained weight, and the mean daily weight gain of the MQL group was significantly greater than that of the control group. There was no significant difference in the average daily water intake between the two groups, but the average daily food intake of rats in the test group was significantly increased. The intake of LF-MQL increased the length of small intestinal villi in rats compared with the control group. HE staining of small intestinal tissue sections showed that the integrity of intestinal tissues was better in both groups, indicating that bovine lactoferrin polypeptide had no adverse effects on the intestinal tract. Moreover, the villi in the MQL group were longer and neater than those in the control group, and the ratio of the length of the villi to the depth of the crypts was elevated, which indicated that LF-MQL improved the intestinal structure of the rats. The analysis of intestinal flora composition showed that LF-MQL had no significant effect on the α-diversity of rat intestinal flora, and the flora abundance and diversity were similar; the effect on the β-diversity of intestinal flora was more significant. Analysis of the intestinal flora showed that the number of ASVs in the intestinal flora of rats in the MQL test group was increased and the relative abundance of some of the beneficial bacteria was increased compared to the control group. COG and KEGG analyses indicated that the intake of LF-MQL could improve the intestinal health of rats by altering the physiological function of the intestinal flora.

## Figures and Tables

**Table 1:** Analysis of change in body weight gain, food intake, water intake, and length of intestinal villi in mice of different groups.

Index	LF-MQL Test Group	Control Group	*p*-Value
Average daily weight gain, g/day/rat	7.54 ± 4.91	6.70 ± 5.40	0.05
Average daily feed intake, g/day/rat	14.44 ± 2.65	11.88 ± 2.86	0.01
Water intake, g/day/rat	43 ± 6.68	43.66 ± 6.81	0.06
Intestinal villus length, (μm)	229.16 ± 34.96	158.94 ± 18.9	3.08 × 10^−7^
Crypt depth, (μm)	149.47 ± 19.13	160.82 ± 13.07	1.0 × 10^−5^
Vh/Cd	2.23	1.61	2.0 × 10^−5^

Note: Values are expressed as mean ± standard deviation.

**Table 2:** Alpha-diversity index of intestinal flora of rats in different groups.

Indes	Con	MQL	*p*-Value
Ace	350.90 ± 37.12	350.73 ± 58.33	0.49
Chao1	350.84 ± 37.25	351.39 ± 58.88	0.49
Shannon	5.91 ± 0.48	5.7 ± 0.25	0.14
Simpson	0.95 ± 0.02	0.94 ± 0.01	0.12

Note: Values are expressed as mean ± standard deviation.

**Table 3:** Relative abundance at gate level.

Taxonomy	LF-MQL Test Group	Control Group	*p*-Value
Bacteroidota	0.726950	0.680897	0.233353
Firmicutes	0.243059	0.300318	0.213803
Proteobacteria	0.026080	0.010023	0.041834
Actinobacteriota	0.002518	0.006496	0.115495
Desulfobacterota	0.000305	0.001262	0.125817
Acidobacteriota	0.000447	0.000285	0.139251
Deferribacterota	0.000264	0.000294	0.44866
Campilobacterota	0.000223	0.00029	0.261275
Fusobacteriota	8.68 × 10^−5^	0.000119	0.261317
Verrucomicrobiota	5.59 × 10^−5^	7.24 × 10^−6^	0.109487
Cyanobacteria	0	9.83 × 10^−6^	0.181609
Gemmatimonadota	5.02 × 10^−6^	0	0.181609

**Table 4:** Relative abundance at the family level, top 10.

Taxonomy	LF-MQL Test Group	Control Group	*p*-Value
Muribaculaceae	0.371438	0.397436	0.189928
Prevotellaceae	0.289351	0.215525	0.0422
Lachnospiraceae	0.116609	0.162811	0.217642
Oscillospiraceae	0.034519	0.032859	0.44549
Rikenellaceae	0.031136	0.036084	0.316364
Ruminococcaceae	0.027072	0.029079	0.430005
Lactobacillaceae	0.023533	0.028163	0.120263
Bacteroidaceae	0.02516	0.022739	0.406802
Clostridia_UCG-014	0.013654	0.019704	0.276281
Enterobacteriaceae	0.022075	0.005709	0.028569
[Eubacterium]_coprostanoligenes_group	0.009276	0.012283	0.307186
Tannerellaceae	0.006787	0.005954	0.310757
Corynebacteriaceae	0.001876	0.00584	0.101063
Peptostreptococcaceae	0.003506	0.004198	0.34562
Marinifilaceae	0.003045	0.003024	0.494248

**Table 5:** Genus-level relative abundance, top 15.

Taxonomy	LF-MQL Test Group	Control Group	*p*-Value
Muribaculaceae	0.370436	0.397215	0.184431
Prevotellaceae_NK3B31_group	0.172412	0.12612	0.024866
Lachnospiraceae_NK4A136_group	0.056388	0.076776	0.210974
Alloprevotella	0.072	0.04766	0.231883
Prevotellaceae_UCG-001	0.044526	0.041354	0.240593
Alistipes	0.031136	0.034555	0.361152
Other	0.02262	0.029657	0.270111
Lactobacillus	0.023533	0.028163	0.120263
Bacteroides	0.02516	0.022739	0.406802
uncultured	0.023617	0.023259	0.480806
Clostridia_UCG-014	0.013654	0.019704	0.276281
Escherichia-Shigella	0.019973	0.003457	0.027653
Ruminococcus	0.014618	0.008208	0.122818
Roseburia	0.004937	0.01782	0.097416
[Eubacterium]_coprostanoligenes_group	0.009276	0.012283	0.307186

**Table 6:** KEGG metabolic pathway Level 1.

Metabolic Pathway	MQL1	MQL2	MQL3	MQL4	MQL5	MQL6	C1	C2	C3	C4	C5	C6
Cellular processes	2,955,558	4,010,865	4,262,654	3,680,410	3,885,557	4,179,252	3,320,333	2,906,437	3,154,340	3,821,559	4,966,342	5,025,938
Environmental information processing	3,244,903	4,811,794	4,905,844	4,208,439	4,509,821	4,971,381	3,720,628	3,311,411	3,504,938	4,423,027	6,411,983	6,986,752
Genetic information processing	7,213,655	8,989,718	9,281,637	8,834,705	8600684	9,251,885	7,429,587	7,413,929	7,622,696	8,947,089	9,316,427	9,463,188
Human diseases	2,734,978	3,358,950	3,515,232	3,305,227	3320353	3,519,992	2,894,728	2,770,719	2,786,328	3,293,662	3,636,118	3,680,817
Metabolism	63,658,556	78,583,729	82,369,378	76,869,077	76362146	80,136,670	68,689,952	64,333,596	66,264,872	77,329,699	83,329,593	84,673,785
Organismal systems	1,615,945	1,985,313	2,106,043	1,974,150	1942786	2,049,555	1,735,522	1,630,816	1,689,005	1,981,239	2,112,324	2,102,006

**Table 7:** KEGG metabolic pathway Level 3, top30.

Metabolic Pathway	MQL1	MQL2	MQL3	MQL4	MQL5	MQL6	C1	C2	C3	C4	C5	C6
Metabolic pathways	15,429,737	18,929,112	19,880,756	18,629,391	18,563,986	19,337,611	16,472,630	15,538,059	16,039,964	18,723,783	20,014,149	20,194,015
Biosynthesis of secondary metabolites	7,463,354	9,272,655	9,690,074	9,032,719	8,939,485	9,417,355	8,095,790	7,545,674	7,845,770	9,085,381	9,837,173	10,038,845
Microbial metabolism in diverse environments	3,357,101	4,252,823	4,442,286	4,100,346	4,130,637	4,366,761	3,655,063	3,416,459	3,502,909	4,129,443	4,629,177	4,783,078
Biosynthesis of amino acids	3,073,189	4,118,172	4,209,507	3,775,596	3,774,655	4,087,748	3,547,199	3,118,051	3,356,129	3,846,264	4,597,747	4,862,194
Carbon metabolism	2,238,321	2,829,463	2,981,325	2,741,393	2,712,035	2,879,239	2,409,229	2,303,759	2,354,952	2,778,438	2,962,728	3,031,856
Ribosome	2,118,851	2,607,021	2,698,334	2,594,942	2,502,940	2,689,567	2,138,647	2,186,165	2,235,884	2,626,235	2,631,844	2,662,131
ABC transporters	1,160,701	1,959,941	1,989,619	1,611,405	1,796,696	1,971,032	1,388,754	1,177,982	1,325,074	1,774,048	2,885,852	3,213,458
Purine metabolism	1,436,815	1,711,882	1,797,918	1,734,558	1,733,851	1,796,387	1,428,764	1,437,211	1,477,336	1,749,379	1,813,901	1,777,253
Two-component system	1,059,817	1,475,987	1,532,395	1,316,051	1,404,770	1,576,018	1,240,754	1,047,867	1,100,929	1,334,907	1,955,673	2,082,410
Pyrimidine metabolism	1,142,547	1,382,701	1,450,269	1,394,894	1,367,490	1,437,669	1,135,529	1,162,905	1,181,258	1,401,324	1,409,765	1,409,582
Glycolysis/gluconeogenesis	1,079,089	1,369,839	1,424,345	1,324,599	1,287,508	1,372,158	1,145,777	1,123,601	1,117,897	1,348,844	1,417,230	1,452,145
Amino sugar and nucleotide sugar metabolism	1,046,722	1,306,386	1,366,782	1,270,613	1,233,497	1,335,872	1,156,662	1,049,073	1,071,897	1,280,964	1,422,852	1,425,239
Quorum sensing	815,163.5	1,215,701	1,232,383	1,065,842	1,116,354	1,248,853	918,547.1	848,799	912,477.7	1,119,812	1,491,175	1,596,853
Aminoacyl-tRNA biosynthesis	898,781.9	1,149,998	1,173,342	1,113,594	1,082,598	1,180,016	921,220.2	932,611.1	962,451.1	1,132,103	1,194,352	1,222,252
Cysteine and methionine metabolism	848,509	1,106,793	1,138,604	1,051,979	1,050,650	1,123,303	899,530.5	872,648.1	911,598.3	1,077,925	1,173,696	1,229,507
Oxidative phosphorylation	901,731.5	1,042,196	1,095,363	1,072,434	1,088,438	1,056,469	906,116.4	902,064.5	929,497	1,082,312	1,003,208	947,952.6
Alanine, aspartate, and glutamate metabolism	899,431.1	1,065,308	1,117,792	1,046,755	1,020,227	1,073,693	989,334.4	893,849.8	918,123.4	1,041,215	1,110,758	1,125,888
Starch and sucrose metabolism	813,831.9	1,106,322	1,130,206	1,006,896	982,614.6	1,087,461	954,093.5	826,498.6	868,234.3	1,039,587	1,302,561	1,372,550
Glycine, serine, and threonine metabolism	837,843.9	1,022,720	1,079,325	1,017,667	1,011,777	1,049,008	859,878.4	854,054.1	873,040.8	1,033,443	1,063,158	1,074,684
Homologous recombination	832,337.9	1,009,861	1,052,572	1,009,588	982,311.5	1,057,878	848,399	837,999.6	866,146.2	1,016,069	1,069,819	1,063,358
Pyruvate metabolism	783,955.2	1,035,829	1,054,142	967,776.2	975,354.3	1,043,539	859,869.8	817,373.6	835,382.4	977,928.7	1,073,606	1,134,189
Carbon fixation pathways in prokaryotes	809,364.1	972,818.3	1,032,192	968,311	953,326.1	992,739.9	873,698.4	822,320	842,435.9	977,513.9	937,709.8	968,111.4
Peptidoglycan biosynthesis	739,578.6	960,897.8	986,802.4	922,080.1	907,552.8	978,318.2	759,381.8	756,641	802,926.8	942,211.9	1,005,872	1,036,490
Mismatch repair	676,460.7	859,143.2	895,405.4	836,604.8	814,121.6	878,819.8	702,223.2	693,774.3	719,436.2	849,215.6	909,672	919,021.5
Methane metabolism	658,710.7	825,541.1	867,974.2	805,664.9	786,575.5	844,293	694,323.5	677,570.1	686,049.9	812,903.2	874,280.3	890,105.1
Phenylalanine, tyrosine, and tryptophan biosynthesis	624,108.7	794,797.7	837,442.2	756,901.5	764,293.7	798,445	690,840.6	610,749.9	681,993.9	769,998.6	867,869.8	871,896.2
Glyoxylate and dicarboxylate metabolism	647,280.3	757,766.8	821,555.1	766,214.6	775,096.1	776,903.9	695,962.5	635,495.4	652,076.6	763,009.3	795,278.7	792,846.1
Pentose phosphate pathway	602,737	801,668.5	838,682.2	747,503.2	754,924	799,153.4	672,060.6	609,100	635,912.1	764,914.7	944,377.7	955,199.2
Fructose and mannose metabolism	638,887.9	758,193.9	814,831	761,140.1	764,835.3	780,800.8	678,310.6	609,602.6	634,022.4	762,440	844,723.9	831,006.9
Galactose metabolism	638,836.9	763,388.1	804,491.3	760,089.2	724,421.6	781,305.3	730,303.7	635,572.5	630,140	752,639	872,389.4	874,061.6

## Data Availability

The original contributions presented in this study are included in the article. Further inquiries can be directed to the corresponding author.
